# The effect of tea fermentation on rosmarinic acid and antioxidant properties using selected *in vitro* sprout culture of *Orthosiphon aristatus* as a model study

**DOI:** 10.1186/2193-1801-2-167

**Published:** 2013-04-16

**Authors:** Dase Hunaefi, Iryna Smetanska

**Affiliations:** 1 Department of Methods in Food Biotechnology, Institute of Food Technology and Food Chemistry, Berlin University of Technology, Königin-Luise Str. 22, Berlin, 14195 Germany; 2 Department of Food Science and Technology, Bogor Agricultural University, Bogor, Indonesia; 3 Department of Plant Food Processing, University of Applied Science Weihenstephan-Triesdorf, Steingruber Str. 2, Weidenbach, 91746 Germany

**Keywords:** Java tea, Jasmonic acid, Yeast extract, Phenolics, Rosmarinic acid, Antioxidant activity, Secondary metabolites

## Abstract

*Orthosiphon aristatus,* an Indonesian medicinal plant, is normally used as a traditional herbal tea. Recently, this plant has begun to attract attention due to its antioxidant properties. However, little is known about tea fermentation effect on antioxidant properties of this plant. Thus, to extend the tea fermentation study, *in vitro* sprout culture of this plant was established as a new feature model. This model plant was selected based on three reasons. Firstly, as a native tropical plant, to grow this plant in sub-tropic area is considered difficult. Secondly, the *in vitro* sprout culture is more genetically stable compared to other types of *in vitro* cultures. Thirdly, results showed that this *in vitro* sprout culture grew faster and produced higher biomass than *in vitro* tissue culture. Both characteristics are important in producing tea leaves. Accordingly, the aim of the current study was twofold. First was to establish high rosmarinic acid line of *in vitro* sprout culture of *Orthosiphon aristatus* by elicitation. Second was to evaluate the effect of tea fermentation on antioxidant properties of this plant. Results showed that yeast extract (5 g/L) elicitation resulted in the highest production of rosmarinic acid. This elicited plant was subjected to partial and full tea fermentation. Results revealed that both tea fermentations decreased the level of rosmarinic acid, total phenolic compounds, flavonoids, and flavonols. These decreases were concomitant with reduced antioxidant activities as measured by 1,1-diphenyl-2-picrylhydrazyl (DPPH) scavenging activity, Trolox equivalent antioxidant capacity (TEAC), and Superoxide dismutase (SOD)-like activity assays. HPLC results showed that the longer the tea fermentation was, the greater reduction rosmarinic acid was found. High correlation value of 0.922 between rosmarinic acid and antioxidant activities was also observed. These results indicated that rosmarinic acid is the major contributor to the antioxidant activities of *Orthosiphon aristatus*. These results may provide useful information, in particular, for the food and pharmaceutical industries in the development of functional foods desiring maximum potential health benefits from *Orthosiphon aristatus*.

## Background

*Orthosiphon aristatus* is an important plant in Indonesian traditional folk medicine. It belongs to *Lamiaceae* family (Ignacimuthu et al., 2003). It has been traditionally used as herbal tea for alternative medicine of various diseases; gout, diabetes mellitus, hypertension, rheumatism, tonsillitis and menstrual disorder and especially those affecting the urinary tract, that is for treating kidney ailments and bladder related diseases (Yam et al., 2010). Surprisingly, there is no published report on the effect of tea fermentation using *Orthosiphon aristatus* plant.

Importantly, *Orthosiphon aristatus* contains high phenolic compounds (Ho et al., 2010). These phenolic compounds are suggested to be the main factors responsible for the medicinal properties of this plant (Ho et al., 2010). One of the most important phenolic compounds in this plant is rosmarinic acid (Akowuah et al., 2005). Rosmarinic acid is an ester of caffeic acid and 3,4-dihydroxyphenyllacticacid. It becomes one of the most targeted phenolic compounds in formulating functional foods and supplements due to its antioxidant activities (Ahamed et al., 2011; Vamanu & Nita, 2013). However, there is virtually no information about the effect of tea fermentation on rosmarinic acid and antioxidant activities of this plant.

The escalating demand of rosmarinic acid has also led many food and pharmacy scientists to find alternative natural sources of this compound (Petersen & Simmonds, 2003). In fact, rosmarinic acid from medicinal plants such as *Orthosiphon aristatus* that contain naturally occurring antioxidants is preferable (Petersen & Simmonds, 2003). *In vitro* plant cultures that accumulate rosmarinic acid have also been proposed for production of this compound (Petersen & Simmonds, 2003). Indeed, the advantage of using *in vitro* plant cultures relies on independently controlling the quality of the products from the environmental factors such as climate and weather (Azeredo, 2009). Additionally, as defined by the European Parliament Directive 2001/18/EC, the *in vitro* culture and its products are not classified as genetically modified organisms (GMOs) and hence are not subject to EU regulations regarding GMOs (OJ, L 106, 17.4.2001, p.1–38) (Georgiev et al., 2010). Thus, establishing *in vitro* plant culture of *Orthosiphon aristatus* and characterizing the rosmarinic acid content and related antioxidant activities may provide useful information, in particular, for food and/or pharmacy scientists.

Accordingly, to extend the fermentation study, *in vitro* sprout culture of *Orthosiphon aristatus* (abbreviated as IOSC) was established. This *in vitro* system was selected based on four reasons. Firstly, as a native tropical plant, to grow *Orthosiphon aristatus* in sub-tropic area is considered difficult. Secondly, this *in vitro* system allows harvest of this plant at any condition regardless of climates condition and seasons (Shevchenko et al., 2010). Thirdly, through this *in vitro* system, rapid growth mass production and sustainable secondary metabolites production can be achieved since this *in vitro* system provides ease control biotic and abiotic stresses on the cultivar (Shevchenko et al., 2010). Finally, compared to other *in vitro* systems, this *in vitro* system is genetically more stable (2008). Consequently, this IOSC is used as a new feature media to facilitate the tea fermentation study.

Aforementioned backgrounds, the main aim of the current study is thus to elucidate the effect of tea fermentation on antioxidant properties using IOSC as a model study. The antioxidant properties in this context consisted of antioxidant activities and components which are determined by a rapid reliable spectrophotometric analysis. The characterization of antioxidant activities is estimated based on 1.1-diphenyl-2-picrylhydrazyl (DPPH) scavenging activity, Trolox equivalent antioxidant capacity (TEAC), and Superoxide dismutase (SOD)-like activity assays. This antioxidant activities’ characterization is followed by the measurement of antioxidant components based on total phenolic compounds, flavonoids, flavonols and rosmarinic acid. The main aim results in three target approaches as follow: (a) to select the high rosmarinic acid line of IOSC by elicitation; (b) to characterize the effect of tea fermentation on antioxidant properties of selected IOSC; and (c) to identify the individual phenolic acids of IOSC and their changing in quantity by tea fermentation.

## Results and discussion

### Selecting of high rosmarinic acid line of IOSC by elicitation for tea fermentation study

This study aimed to evaluate the effect of tea fermentation on rosmarinic acid and antioxidant activities of IOSC. However, as described in the background, firstly, we developed the high rosmarinic acid line of IOSC by elicitation. The effectiveness of the *in vitro* sprout culture has been tested in our laboratory (Shevchenko et al., 2010) and it was demonstrated in our results by using *Orthosphon aristatus* plant. Preliminary research showed that *in vitro* sprout culture of *Orthosiphon aristatus* was capable of developing faster and had higher biomass compared to that of *in vitro* tissue culture. Both characteristics, growing faster and producing higher biomass, are important in producing tea leaves. The main difference between *in vitro* sprout culture and *in vitro* tissue culture of *Orthosiphon aristatus* is an aggregate state of the nutrient medium. The nutrient medium of the *in vitro* sprout culture, regardless of nutrient content and sucrose concentration on it, is always liquid. Another difference in the *in vitro* sprout culture is that it is always agitated. Further information about *in vitro* sprout culture was explained by Sevchenko et al. (2010). Data of two-week IOSC behaviour subjected to elicitation are depicted in Table 1.

**Table 1:** **Data of two-week IOSC behaviour subjected to elicitation**

Sample group	IOSC Length (cm)	Number of leaves	Number of roots	Fresh weight (FW) (g)
Control	5.02 ± 0.58	9.40 ± 2.88	4.40 ± 1.14	6.29 ± 1.44
JA	3.88 ± 0.94	5.80 ± 1.30	3.80 ± 2.17	4.23 ± 0.86
YE	3.10 ± 0.80	5.20 ± 1.30	3.60 ± 1.95	5.40 ± 1.18

Data reported as average ± SD with n = 5.

Control = MS medium; JA = MS medium with jasmonic acid; YE = MS medium with yeast extract.

**Figure 1:**
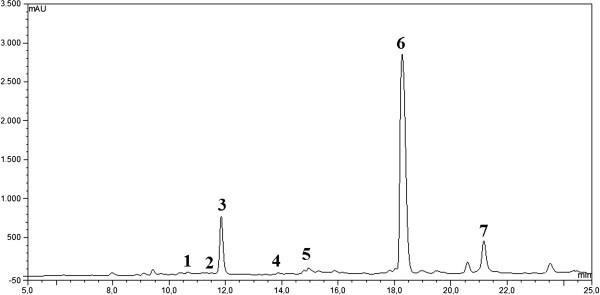
**Typical HPLC chromatogram of IOSC extract subjected to elicitation: 1.** vanillic acid; 2. chlorogenic acid; 3. caffeic acid; 4. *p*-coumaric acid; 5. sinapic acid; 6. rosmarinic acid; and 7. cinnamic acid as internal standard.

HPLC analysis of two-week freshly harvested IOSC is presented in Figure 1. Several identified phenolic acids, vanilic, chlorogenic, caffeic, *p*-coumaric, sinapic and rosmarinic acid, were eluted in the chromatogram (Figure 1). Rosmarinic acid was found to be the major phenolic of all identified phenolic constituents in IOSC extract by HPLC analysis. It was followed by caffeic acid as the second major phenolic acid in IOSC extract. This finding was in agreement with the report on the analysis of this plant by Sumaryono et al. (1991b).

In response to elicitation, an increased in rosmarinic acid was noticed for all samples compared to the control (Table 2). The basal level of rosmarinic acid content in the control sample was 2.89 ± 0.86 mg/g DW, meanwhile in IOSC samples elicited with jasmonic acid was 3.75-folds higher (10.83 ± 0.27 mg/g DW) (Table 2). Nevertheless, the highest elicitation effect was seen in IOSC samples treated with yeast extract resulting in 4.95-folds rosmarinic acid content (14.28 ± 0.64 mg/g DW) in comparison to that of the control (Table 2). These results showed that the elicitors’ jasmonic acid and yeast extract were very effective in inducing the production of rosmarinic acid in IOSC. These results were consistent with those previously described by Ogata et al. (Ogata et al. 2004) who found out that an addition of yeast extract and methyl-jasmonate enhanced the accumulation of rosmarinic acid rapidly in the *in vitro* culture of *Lithospermum erythrorhizon*.

**Table 2:** **Individual response of phenolic acids subjected to elicitation**

Phenolic acids	Chemical structure	Control (mg/g DW)	Fold of control	
			JA	YE
rosmarinic acid		2.89 ± 0.86	3.75	4.95
(MW: 360.31 g/mol)				
vanillic acid		0.31 ± 0.03	2.80	3.72
(MW: 168.14 g/mol)				
chlorogenic acid		0.07 ± 0.01	1.10	1.90
(MW: 354.31 g/mol)				
caffeic acid		0.58 ± 0.02	1.30	3.46
(MW: 180.16 g/mol)				
*p*-coumaric acid		0.04 ± 0.03..	2.40	3.50
(MW: 164.16 g/mol)				
sinapic acid		0.14 ± 0.04	2.50	1.82
(MW: 224.21 g/mol)				

Reported values are the means ± S.D. (n = 5); MW = Molecular Weight.

Control = MS medium; JA = MS medium with jasmonic acid; YE = MS medium with yeast extract.

Moreover, Kim et al. (2001) reported that the stimulation of rosmarinic acid biosynthesis in *Agastache rugosa* O. Kuntze was very successful in response to the addition of yeast extract in the media which could raise rosmarinic acid content up to 5.7-folds compared to the non-elicited suspension cells. The increase in rosmarinic acid content by yeast extract treatment, as reported here, has been suggested to be correlated with the increase in phenylalanine ammonia-lyase (PAL) activity, indicating the important role of PAL activity in the regulation of rosmarinic acid biosynthesis (Mizukami et al., 1992; Sumaryono et al., 1991a).

Another explanation of increasing rosmarinic acid in IOSC is that *Orthosiphon aristatus,* like other plants, carry out an elicitor-mediated defence response by inducing phenylpropanoid pathway resulting in producing more rosmarinic acid. As a result, yeast extract elicitor was found to be the best strategy for rosmarinic acid production in comparison to jasmonic acid as equally reported in other studies (Mizukami et al., 1992; Sumaryono et al., 1991a). Therefore, IOSC with yeast extract elicitor was used to study the effect of tea fermentation in IOSC.

In addition, the responses of other phenolic acids are provided in Table 2. Yeast extract elicitation, however, caused an increased in vanillic, caffeic acid and *p*-coumaric acid by 3.72, 3.46 and 3.50 fold of control, respectively. In other plant species, *Mallus domestica* cell culture, *p*-coumaric acid and chlorogenic acid were more responsive to the yeast extract elicitation compared to jasmonic acid elicitation (Cai, 2012). In agreement with that report by Cai (2012), the higher increase of *p*-coumaric, caffeic and vanillic acid due to yeast extract elicitation, in comparison to jasmonic acid one, was also demonstrated. These observations indicated that yeast extract elicitation, not only increased rosmarinic acid content but also enhanced the production of other phenolic acids. This observable fact strengthened the chosen of yeast extract-IOSC for tea fermentation study.

### Evaluating the effect of tea fermentation on rosmarinic acid and antioxidant activities using selected IOSC

Tea is known to be one of the most favourite beverages in the world. Actually, the term ‘tea fermentation’ in this context refers to natural browning reactions induced by oxidative enzymes in the cells of tea leaves (Kuo et al., 2011). The effect of tea fermentation on antioxidant properties of IOSC are shown in Table 3.

**Table 3:** **The effect of tea fermentation on antioxidant properties of IOSC**

Antioxidant Properties of tea IOSC*	UFT^a^	PFT^b^	FFT^c^
DPPH (% AA: Antioxidant activity)	18.38 ± 1.82	13.27 ± 1.24	6.08 ± 1.33
TEAC (mM/g DW Trolox equivalent (**TE**))	1.35 ± 0.15	0.95 ± 0.07	0.50 ± 0.15
SOD-like activity (%)	46.75 ± 3.77	44.38 ± 2.85	39.28 ± 4.65
Total flavanoids (mg QE/g DW)	2.66 ± 0.16	2.24 ± 0.08	1.73 ± 0.03
Total flavonols (mg QE/g DW)	2.00 ± 0.09	1.81 ± 0.11	1.86 ± 0.12
Rosmarinic acid content (mg/g DW)	1.13 ± 0.25	0.70 ± 0.09	0.10 ± 0.05

*UFT*, unfermented; *PFT*, partial fermented; and *FFT*, full fermented.

*Reported values are the means ± S.D. Means denoted the same letter for each set of data did not significantly differ at *p* <0.05 according to Tukey HSD test.

There are several published reports about tea fermentation (Ariffin et al., 2011; Heong et al., 2011; Kim et al., 2011; Takano-Ishikawa et al., 2012; Xu et al., 2011), but most of them are focused on flavonoids content. Besides, those published reports on tea fermentation are used the common popular tea plants which are *Camellia sinensis* and *Centella asiatica*. Surprisingly, all those published reports employ a terrestrial plant system as a media for studying the effect of tea fermentation. We have not yet come across any published reports describing the effect of tea fermentation using an *in vitro* plant system. Therefore, to the best of our knowledge, this is one of the first studies where *in vitro* sprout culture of *Orthosiphon aristatus* is used to extend the tea fermentation study focused on rosmarinic acid and antioxidant activities.

Black (full-fermented) and oolong (semi-fermented) tea processing methods of *Camellia sinensis* were used to produce IOSC tea leaves with two-hour withering process at 30°C. Hot water infusion method for normal house tea preparation method was adopted to characterize of antioxidant properties in the IOSC tea. Although there are many other better options to extract antioxidants in IOSC, for example using methanol and ethanol as solvents for extraction, however, the safety of both solvents is remained questionable (Mohdaly et al., 2010). For that reason, the hot water infusion method is chosen with the advantage of providing real condition to the readers of how a drinking tea from this plant is made without compromising the safety of the solvent.

In general, the results showed that the effect of tea fermentation resulted in the diminution of antioxidant activities of IOSC as measured by three antioxidant assays (Table 3). Note that in our study, we used and compared three antioxidant assays to characterize the potential antioxidant activities of IOSC. It should be kept in mind that the antioxidant activities cannot be measured directly but rather by the effects of the antioxidants in controlling the extent of oxidation (Fernandez-Orozco et al., 2007). On the one hand, many of the antioxidant assays show great differences. On the other hand, they can be used to characterize the potential of health functionalities of raw material and their evolution during processing such as tea fermentation (Fernandez-Orozco et al., 2007).

It has been suggested that antioxidant components may react differently to different antioxidant assays (Debnath et al., 2011). For instance, Wang et al. (1998) found that some compounds, which have 2,2^′^-azinobis-(3-ethylbenzothiazoline-6-sulfonate) (ABTS) radical scavenging activity, may not show 1,1-diphenyl-2-picrylhydrazyl (DPPH) radical scavenging activity. Since fermentation may alter the composition of antioxidants compounds, thus, one antioxidant assay is not sufficient for the complete assessment of those activities.

Two of the most used methods are DPPH scavenging activity and TEAC assays. DPPH free radical scavenging activity is based on the ability of the presence antioxidant compounds to decolorize DPPH reagent. The presence of antioxidants in the mixed solution reacts with DPPH and reduces the number of DPPH free radicals to the number of their available hydroxyl groups indicated by discoloration of the solution (Mohdaly et al., 2010). Whereas, TEAC method is based on the extent of de-coloration as the indicator of inhibition of ABTS by the availability of antioxidants compounds. Moreover, it is calculated relative to the reactivity of Trolox as standard under the same conditions (Re et al., 1999). To complete those antioxidant activities assessments, it was considered important to use a model assay that mimics our body system such as superoxide-dismutase (SOD)-like antioxidant assay (Fernandez-Orozco et al., 2007). Basically, the determination of SOD-like activity permits the assessment of superoxide anion scavenging activity when a xanthine and xanthine oxidase system is used to originate superoxide radicals (Fernandez-Orozco et al., 2007). Therefore, those three antioxidant assays were determined and compared in our tea fermentation study.

As can be seen from Table 3 the DPPH radical scavenging activity of UFT was 18.38 ± 1.82% and it decreased to the level of 13.27 ± 1.24% in PFT. More dramatic decrease was suffered in FFT samples which having the level DPPH scavenging activity only 6.08 ± 1.33%. The lessening result of DPPH scavenging activity due to tea fermentation was in agreement with recent investigation on DPPH scavenging activity of *Camellia sinensis* reported by Takano-Ishikawa et al. (2012).

Interestingly, in our results, a similar trend was also found for TEAC and SOD-like antioxidant activities. Using TEAC method, the antioxidant activity TEAC value of UFT was 1.35 ± 0.15 mM/g DW TE and it decreased to 0.95 ± 0.07 and 0.52 ± 0.15 mM/g DW TE for PFT and FFT, respectively (Table 3). In case of SOD-like antioxidant activity, similar finding to DPPH and TEAC was also observed. The gradual lessening of percentage antioxidant activity was demonstrated by using this SOD-like antioxidant activity assay. These decreases in antioxidant activities during tea fermentation could be associated with reduced of antioxidant components in IOSC (Table 3).

Table 3 also highlights the antioxidant compounds in IOSC extracted by hot water infusion method. Total phenolics, flavonoids, flavonols and rosmarinic acid content of unfermented IOSC samples (UFT) were 73.81 ± 11.24 mg GAE/g DW, 2.66 ± 0.16 mg QE/g DW, 2.00 ± 0.09 mg QE/g DW, and 1.13 ± 0.25 mg/g DW, respectively. The level for all antioxidant components was much higher in UFT compared to those in the partial (PFT) and full (FFT) fermentation of IOSC (Table 3). In the partial fermented of IOSC (PFT), the level of the estimated total phenolics, flavonoids, flavonols and rosmarinic acid were 69.37 ± 10.08 mg GAE/g DW, 2.24 ± 0.08 mg QE/g DW, 1.81 ± 0.11 mg QE/g DW and 0.70 ± 0.09 mg/g DW respectively. Meanwhile, there was a further decrease in the FFT of IOSC with the level of total phenolics, flavonoids, flavonols and rosmarinic acid being 42.70 ± 7.56 mg GAE/g DW, 1.73 ± 0.03 mg QE/g DW, 1.86 ± 0.12 mg QE/g DW, and 0.10 ± 0.04 mg/g DW respectively.

Takano-Ishikawa et al. (2012) observed that full-fermented tea of *Camellia sinensis*, known as black tea, had lower total phenolics ranging from 74.6 to 119.4 mg GAE/g DW compared to unfermented tea of *Camellia sinensis* (green tea) which was from 98.1 to 154.6 mg GAE/g DW. They further reported that the power of DPPH radical scavenging activity showed a very similar trend to the results obtained from total phenolics. They suggested that the higher antioxidant activity in unfermented tea of *Camellia sinensis* (green tea) is explained by the higher total phenolics compared to those of the fully-fermented tea of *Camellia sinensis* (black tea). Similarly, Nshimiyimana & He (2010) reported the antioxidant activity of full-fermented tea of *Camellia sinensis* (black tea) has significantly lower than the antioxidant activity in unfermented one (green tea).

Meanwhile, Satoh et al. (2005) concluded that the oxidation of the polyphenols in green tea during fermentation may greatly contribute to the difference in antioxidant activity among green (un-fermented), oolong (partial-fermented), and black (full-fermented) teas. They explained that the green tea which was unfermented had the highest antioxidant activity (Satoh et al., 2005). This phenomenon was confirmed in our experiment using *in vitro* sprout culture of *Orthosiphon aristatus* as a media for tea fermentation study.

Our results here reported were also in agreement with recent tea fermentation study on *Centella asiatica* by Ariffin et al. (2011). They explained that tea fermentation induces polyphenols oxidation reactions by the enzyme of polyphenols oxidase which resulted in the breakdown of phenolic compounds as main contributor to the antioxidant activities. The tea fermentation time is affected the level of phenolic compounds (Heong et al., 2011). Heong et al. (2011) reported that prolonged fermentation time caused more severe damage of polyphenols in *Centella asiatica* tea as compared to partial fermentation. This damage resulted in a drastic decrease of the potential antioxidant activities of *Centella asiatica*. In agreement with that study by Heong et al. (2011), we also observed that the longer fermentation in full-fermented IOSC had lower level of total phenolic compounds and antioxidant activities than in partial-fermented IOSC. Thus, the results implies that time for tea fermentation is quite crucial which may affect the level of phenolic compounds and potential antioxidant activities of the tea plants.

Phenolics, as one of the main components in IOSC, are reactive compounds. They can be degraded and polymerized through both enzymatic and non-enzymatic reactions during tea processing of IOSC. Concerning the enzymatic reactions, these phenolics can be oxygenated and also damaged by various enzymes for example; polyphenol-oxidases (PPO), peroxidases, glycosidases and esterases, that are released when plant cells are broken down (Shoji, 2007). The damage of the phenolics in fermented tea due to enzymatic processes is indicated by the occurrence of black pigments in tea leaves (Shoji, 2007). This phenomenon was also detected in our results.

With regard to the non-enzymatic reactions during tea fermentation processes, the degradation of phenolics are observed by the presence of tea flavour compounds (Shoji, 2007). It has been known that phenolics can also react among themselves and with other compounds. Such reactions include the high molecular weight artificial polymeric compounds generated during tea processing, via non-enzymatic reactions, presenting tea flavor compounds (Shoji, 2007).

Despite the fact that phenolics are degraded during tea fermentation process that lead to the decreasing of potential antioxidant activity as demonstrated in our results, these reactions are, however, important for producing the colour tones and strengths of tea-beverage aroma (Shoji, 2007). In other words, these enzymatic and non-enzymatic reactions during tea fermentation are indispensable in producing black (full-fermented) and oolong tea (partial fermented) tea (Shoji, 2007).

Furthermore, in comparison to the freshly harvested IOSC leaves, the reduction in antioxidant properties was noticed in the control sample which did not undergo fermentation but was only subjected to drying process at 80°C in vacuum oven for 12 hours. For instance, the total phenolics, flavonoids and flavonols of water extract in freshly harvested IOSC elicited with yeast extract was 82.33 ± 2.08 mg GAE/g DW, 2.96 ± 0.07 and 2.59 ± 0.16 mg QE/g DW, respectively, whereas after drying (control for tea fermentation), the total phenolics, flavonoids and flavonols decreased to 73.81 ± 81 mg GAE/g DW, 2.66 ± 0.16 and 2.00 ± 0.09 mg QE/g DW, respectively.

**Figure 2:**
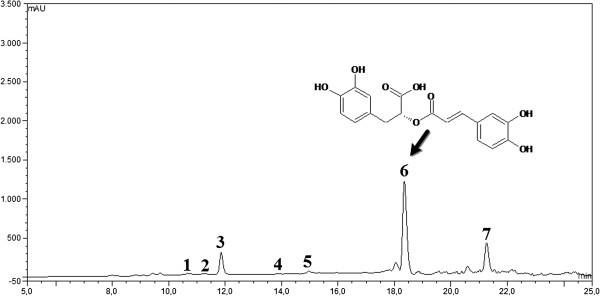
**Typical HPLC chromatogram of IOSC extract subjected tea fermentation: 1.** vanillic acid; 2. chlorogenic acid; 3. caffeic acid; 4. *p*-coumaric acid; 5. sinapic acid; 6. rosmarinic acid; and 7. cinnamic acid as internal standard.

Additionally, individual phenolic acids after tea fermentation were then analyzed by HPLC method. The typical HPLC chromatogram of IOSC after tea fermentation is presented in Figure 2. Meanwhile, the quantification of identified phenolic acids and their changes during the tea fermentation of IOSC are depicted in Table 4.

**Table 4:** **Individual response of phenolic acids to the partial and full fermentation of IOSC tea**

Phenolic acids	UFT (mg/g DW)	% decrease	
		PFT	FFT
rosmarinic acid	3.38 ± 1.07	47.1	63.9
vanillic acid	0.82 ± 0.03	46.2	57.3
chlorogenic acid	0.26 ± 0.04	34.8	51.1
caffeic acid	1.08 ± 0.05	42.4	61.7
*p*-coumaric acid	0.27 ± 0.03	26.1	31.6
sinapic acid	0.18 ± 0.04	27.3	31.4

Appraising the effect of tea fermentation in IOSC, the additional derivatives peaks of phenolic acids were observed. This indicated several processes involved during tea fermentation led to breakdown of phenolic acids. Unfortunately, due to lacking of phenolic acids derivative standards, the unknown peaks remained unidentified. Although many phenolic acids have distinctive UV-spectra (Mewis et al., 2011), we encountered difficulties in analyzing some of them, thus, more work and studies are needed to fully elucidate these changes.

It should also be informed that the method used to produce unfermented tea (UFT) of IOSC was adopted from the processing method of green tea in *Camellia sinensis*. To produce the UFT, IOSC was subjected to heat treatment in vacuum oven at 80°C and 12 hours. Notably, a significant difference in rosmarinic acid content was observed between fresh IOSC elicited with yeast extract and unfermented tea of IOSC (Tables 2 and 4). The levels of rosmarinic acid in the yeast extract fresh IOSC with freeze-drying treated was (14.28 ± 0.64 mg/g DW) which was four times higher than in unfermented tea (UFT, 3.38 ± 1.17 mg/gDW). The results clearly showed that heat treatment involved in producing tea leaves resulted in a sharp decrease of rosmarinic acid level. This reduction was first noticed in UFT (unfermented tea of IOSC).

Similar results were reported by Fletcher et al. (2005) who showed that heat treatment at only 30°C significantly reduce the content of rosmarinic acid and phenolic compounds in spearmint (*Mentha spicata* L). The results showed that rosmarinic acids as similar to other phenolics acids are prone to high temperature. This characteristic might be explained by the structure of rosmarinic acid. This phenolic acid has double bonds (Figure 2) and these double bonds make rosmarinic acid very reactive to heat treatments (Fletcher, et al., 2005).

As for the tea fermentation of oolong (PFT) and black tea (FFT) processing of IOSC leaves, the level of rosmarinic acid was sharply reduced in both PFT (1.79 ± 0.35 mg/g DW) and FFT (1.22 ± 0.12 mg/g DW) compared to that in UFT (3.38 ± 1.17 mg/g DW) (Table 4). This finding is in agreement with current investigation on rosmarinic acid content in *Centella asiatica* tea carried out by Ariffin et al. (2011). They used 80% methanol to extract phenolic cids in *Centella asiatica* and found that tea fermentation causes a reduction in rosmarinic acid. They further reported that the rosmarinic acid level decrease significantly from 282 ± 42 μg/g DW in the unfermented to 158 ± 40 μg/g DW in partial fermented *Centella asiatica* leaves and there was a further reduction to 58 ± 1 μg/g DW in the full fermented *Centella asiatica* tea leaves.

Of other phenolic acids in IOSC, the trend was similar to rosmarinic acid result, that tea fermentation resulted in diminishing of the phenolic acids with the percentage of decrease ranging from 26.1% to 57.3%. These results also imply that heat treatment and reaction involved during the tea processing of IOSC may lead to the destruction of phenolic acids. Level of the reduction appeared to be heavier when full tea fermentation of IOSC was employed compared to partial fermentation. These results agree with those obtained on phenolic acids, gallic and chlorogenic acid of *Centella asiatica* by Ariffin et al. (2011). They reported that full tea fermentation causes the reduction of gallic acid for more than 70%. Similarly, Heong et al. (2011) reported that the longer tea processing time in full-fermented *Centella asiatica* resulted in higher reduction rate of phenolic compounds compared to partial-fermentated one.

Despite the results that tea fermentation of IOSC was deleterious to phenolic acids and counterproductive for capturing high antioxidant activities of the IOSC. However, since the herbal-tea market and demand of rosmarinic acid have grown extensively due to an increased of the health awareness, therefore, these results may provide fundamental and practical information for the herbal-tea industries and consumers in considering the process employed to obtain maximum potential antioxidant activities of *Orthosiphon aristatus*.

### Correlation analysis between the experimental variables

In regard of acquiring a holistic approach to the antioxidant activities and respective partial chemical constituents, a Pearson correlation matrix between all these variables was performed and presented in Table 5.

**Table 5:** **Pearson correlation coefficients matrix between these entire variable**

Pearson correlation coefficients, N = 9						
Prob > |r| under H0: Rho = 0						
	DPPH	TEAC	TPC	Flavanoids	Flavonols	RA
**DPPH**	1	*0.0073*	*0.0157*	*0.0013*	*0.0060*	*0.0304*
**TEAC**	**0.81595**	**1**	*0.0050*	*0.0040*	*0.0012*	*0.0004*
**TPC**	**0.76774**	**0.83614**	**1**	*0.0004*	*<0.0001*	*<0.0001*
**Flavanoids**	**0.88932**	**0.84659**	**0.92124**	**1**	*0.0001*	*0.0025*
**Flavonols**	**0.82642**	**0.89214**	**0.96303**	**0.94512**	**1**	*0.0001*
**RA**	**0.71486**	**0.92231**	**0.96051**	**0.86609**	**0.94726**	**1**

The top right and bottom left diagonals respectively represent the *p*-values and correlation coefficients.

The correlation coefficients were written in bold, while the *p*-values were written in italic.

*TPC*, Total phenolic compounds.

*RA*, Rosmarinic acid.

Results showed that decreasing antioxidant activities was also followed by the decrease in total amount of phenolics, flavonoids, flavonols and rosmarinic acid (Table 5). It has been suggested that the antioxidant activities of plant extracts are mainly due to phenolic compounds (Mohdaly et al., 2010). This positive correlation was also demonstrated in our results.

However, Table 5 also highlights the highest correlation value of 0.922 was found between antioxidant activities and rosmarinic acid content, followed by that with total flavonols, flavonoids and lastly for total phenolic compounds. This correlation results suggested that rosmarinic acid content in IOSC extract had a high contribution and was more likely to be responsible for antioxidant activities of IOSC extract. This phenomenon is also reported by Tepe (2008). Tepe (2008) reported that rosmarinic acid is responsible for the antioxidant activities of Salvia plant.

The high correlation result between rosmarinic acid and antioxidant activities as observed in our result might be justified by several reasons. The first possible reason is that not all phenolic acids have the same antioxidant activities (Soobrattee et al., 2005), and rosmarinic acid is one of the most strongest antioxidants among other phenolic acids (Verma et al., 2009); (2). The second one is that the difference between structure, type and number of substituent on the phenyl ring in the structure of phenolic acids causes the difference in reactivity and quenching ability of the free radicals (Lien et al., 1999). The structures of identified phenolic acids in IOSC are illustrated in Table 2. Compared to other identified phenolic acids in IOSC, rosmarinic acid contains more double bonds in which can be explained its high reactivity to free radical. Finally, rosmarinic acid has antioxidant activity stronger than that in vitamin E (Park et al., 2008) that may also support this correlation result.

Concerning tea fermentation, this correlation also supported the results that this process negatively regulates rosmarinic acid biosynthesis in IOSC and causes a breakdown of this compound. This breakdown of rosmarinic acid may cause the reduction of potential antioxidant activities as determined by DPPH and TEAC assays. In addition, the results suggested that to obtain optimum antioxidant activities of this plant, consuming freshly of *Orthosiphon aristatus* is recommended. However, with regard to preservation, storage, convenience, and sensory aspects, tea fermentation products of this plant would also be preferable. Importantly, these results should be, therefore, considered by food industries, especially by tea manufacturers, in the development of functional foods desiring maximum potential health benefits from *Orthosiphon aristatus*. Certainly, further research on sensory evaluation and degradation products of phenolic compounds in *Orthosiphon aristatus* is needed.

## Conclusions

As a result of elicitation treatment to develop high rosmarinic acid line of IOSC, it was demonstrated that yeast extract elicitation increased the level of rosmarinic acid compared to the control. Therefore, this yeast extract elicited line of IOSC was used for tea fermentation study. From the tea fermentation study using selected IOSC as a model, the results showed that tea fermentation did affect the antioxidant activities and compounds. Tea fermentation decreased the antioxidant activities of IOSC. These decreases were associated with reduced antioxidant compounds of IOSC: total phenolic compounds, flavonoids, flavonols and rosmarinic acids. HPLC results also showed that the longer fermentation was, the greater reduction rosmarinic acid and other phenolic acids content were found. The result of correlation analysis indicated that rosmarinic acid was the major contributor to the antioxidant activities in IOSC. Although further research is required to identify the products’ degradation of the bioactive compounds in *Orthosiphon aristatus*, these present results may provide useful information, particularly, for the food industries and consumers to take into account when they need to obtain optimum potential health benefits from *Orthosiphon aristatus*.

## Materials and methods

### Materials

#### Plant materials

*Orthosiphon aristatus* with article number of 600646 was purchased online from Pflanzenversand Hans-Günter Röpke (http://www.pflanzenkindergarten.de). This was used for the establishment of *in vitro* sprout culture.

#### Culture media

Agar Cell culture media and MS medium (Merck, Darmstadt, Germany), Oxoid Yeast Extract (Code L21) (Unipath LTD, Hampshire, England), (±) Jasmonic acid (Product of Israel, Plant cell culture tested, C_12_H_18_O_3_) (Sigma-Aldrich Chemie GmbH, Steinheim, Germany), and other materials were analytical and plant cell culture tested grades.

### Chemicals for analysis

Gallic acid, ABTS (2,2’-azino-bis (3-ethylbenzthiazoline-6-sulphonic acid)) and the standards of phenolic acids were obtained from Sigma-Aldrich Chemie GmbH (Steinheim, Germany); Quercetin Dihydrate (C_15_H_10_O_7_ · 2H_2_O ~99% HPLC) was from Fluka, Biochemika (Neu-Ulm, Germany). All used chemicals were analytical HPLC grade.

## Methods

### Establishment of IOSC

The establishment procedure of IOSC was based on previous report of Shevchenko et al. (2010) with liquid MS medium modification as follow: MS medium (control), MS with jasmonic acid (100 μM) (JA), and MS medium with yeast extract (5 g/L) (YE). Briefly, Stems from the acclimatized mother plant were cut into 2.5 – 5 cm and sterilized in 70% ethanol for 20–30 seconds. They were then transferred into 10% sodium hypochlorite for 5 min, and washed three times in sterile water before being sown into 250 ml Erlenmeyer flasks on Murashige and Skoog medium (MS; Murashige and Skoog, 1962) for 4 weeks. Stems of sterile plants were cut into 1 – 1.5 cm pieces (±100 mg fresh weight/L) and placed in 250 ml Erlenmeyer flasks containing 50 ml liquid MS medium. IOSC were transferred to a new medium every four weeks and maintained at 25 ± 2°C on a rotary shaker under light irradiation. The best line of IOSC which was the third generation from the first establishment was used for this research. Two-week IOSC was harvested and the length, biomass (fresh weight of the plant), number of leaves and number of roots were monitored.

### Tea fermentation of IOSC

Black and oolong tea processing method were used to produce IOSC fermented leaves. The two weeks of IOSC were harvested, cleaned with distilled water and were withered at 30°C vacuum oven (Heraeus Instruments VT 6025 Vacuutherm Vacuum Oven, Hanau, Germany) for 2 hours. The IOSC leaves were allowed to undergo partial fermentation in incubator (Heraeus, Hanau, Germany) with temperature of 37°C for 2 hours (Partial-fermented tea: PFT). For full fermentation (Full-fermented tea: FFT), the similar procedure was repeated but the fermentation time was prolonged to 24 hours. The fermented teas were exposed to drying process using vacuum oven at 80°C to inactive polyphenol oxidase (PPO) for 12 hours until the moisture content was less than 6.5%. The dry IOSC teas were kept in air tight container and stored in −20°C for further analysis. As a control, green tea (unfermented tea) processing method which is unfermented tea was adopted. In brief, the fresh two weeks of IOSC leaves were harvested and also subjected to drying at vacuum oven for 12 hours (unfermented tea: UFT).

### Sample extraction for tea fermentation samples

The dried-IOSC tea leaves, UFT, PFT, and FFT were manually twisted and torn for around 5 minutes. Twisted tea leaves were weighed one gram and placed into a beaker glass. Hot distilled water (100°C, 100 ml) was then added and allowed to infuse the leaves for 10 min. The infusions were filtered through vacuum filter with Whatman paper No. 1 for analysis.

### Antioxidant properties of the IOSC

#### DPPH free radical scavenging assay

The free radical scavenging activity of the extracts, based on the scavenging activity of the stable 1,1-diphenyl-2- picrylhydrazyl (DPPH) free radical was conducted as previously described by Mohdaly et al. (2010) with a few modifications. Shortly, 0.001 mol/L DPPH stock solutions was prepared by dissolving 22 mg of DPPH in 50 ml of methanol and stored at −20°C until use. For the measurement, 6 ml of the stock solution was diluted with 100 ml of methanol to an absorbance of 0.800 ± 0.02 at 515 nm. An aliquot of 50 μl of extracts was added to 2 ml of the freshly prepared DPPH working solution and was vortexed for 30 seconds. The absorbance at 515 nm was recorded every 5 minutes until 30 minutes using spectrophotometer (PerkinElmer Precisely Lambda 25 UV/VIS Spectrometer, PerkinElmer, MA, USA) against the control with no added extracts. The percentage inhibition activity as relative antioxidant activity was calculated based on following formula:

where AC is the absorbance of the control, and AS is the absorbance of the samples.

### Trolox equivalent antioxidant capacity (TEAC)

**Figure 3:**
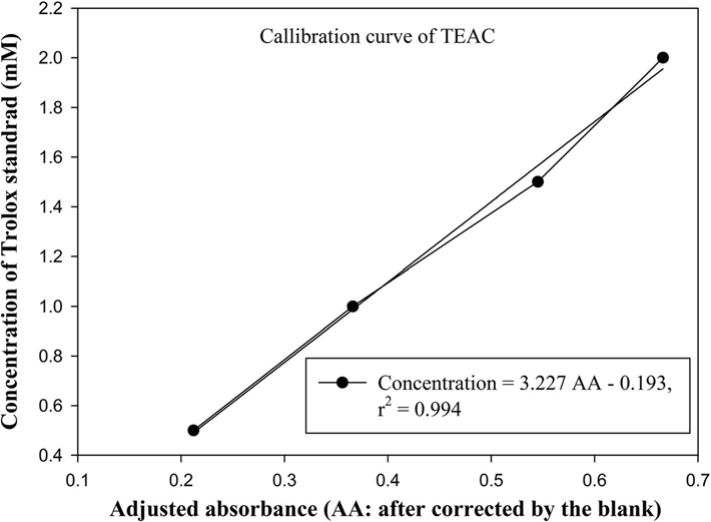
**Trolox® standard calibration curve of OD**_**734nm**_**versus concentration of Trolox in mM.**

TEAC assay was performed according to the protocol as previously described by Re et al. (Re et al., 1999). 38.43 mg ABTS (7 mM) and 6.90 mg K_2_S_2_O_8_ (2.45 mM) were accurately weighed and mixed in dark-brown glass (covered with alumunium foil) with an aliquot 10 ml distilled water. This ABTS^●+^ -stock solution was kept standing in the dark for 12 – 24 hours in order to react completely and stable. The working solution was prepared by mixing 1 ml of ABTS^●+^ -stock solution with 100 ml of ethanol (p.a.) to obtain an absorbance of 0.700 ± 0.02 at 734 nm named as A1. Trolox stock solution was prepared by accurately weighed 12.53 mg Trolox® in aliquot 5 ml ethanol (p.a.). The ethanolic-concentrated Trolox stock solution 0.01 mol/L was then exactly pipetted of 250, 500, 750, and 1000 μl and was diluted with ethanol (p.a.) making the total volume of 5 ml. These concentrations of amounted from 0.5 to 2.0 mmol/L Trolox®. A 20 μl aliquot of extracts, standard Trolox® solutions and ethanol as a blank (A0) were added to 1 ml of ABTS^●+^ -working solution and the mixture was vortexed for 30 seconds. The absorbance of 734 nm using spectrophotometer (Jenway System, UK) and a semi-cuvet were used for determination. The absorbance was measured at 734 nm after interacting with samples soution for exact 6 minutes as recomended by Re et al. (Re, et al., 1999). The decrease in absorption at 734 nm after the addition of the reactant was used to calculate the TEAC value. The difference between absorbance of working solution and blank called ΔA1 [ΔA1 = A1 - A0]. And the difference among the results of the working solution, blank and the samples gave ΔEsample [ΔEsample = A1 - Asamples (extracts/standards) - ΔA1], the value used for further calculations. A Trolox® standard calibration curve of OD_734nm_ versus concentration of Trolox in mM was constructed. The final results were expressed as mM Trolox equivalent (TE) per gram dry weight of samples (mM/gDW TE). The antioxidant activity of samples’ extracts was calculated using the equation in Figure 3.

### Superoxide Dismutase (SOD)-like activity

SOD-like activity was measured according to the previously described method by Debnath et al. (2011). The solution was prepared by mixing 0.2 mL of the IOSC extracts, 3 mL of the Tris–HCl buffer (50 mM Tris[hydroxymethyl]aminomethane + 10 mM EDTA, pH 8.5), and 0.2 mL of 7.2 mM pyrogallol. The solution was allowed to stand for 10 min at 25°C. The pyrogallol oxidized was measured at 420 nm using a spectrophotometer after the reaction was terminated by adding 0.1 mL of 1.0 M HCl solution. Percentage of SOD-like antioxidant activity was calculated as follow:

### Total phenolic compounds (TPC)

**Figure 4:**
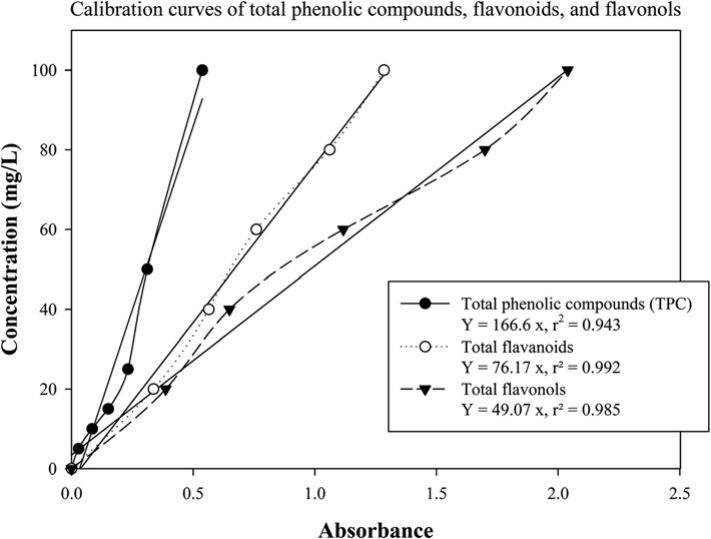
**Calibration curves to estimate total phenolic compounds, flavonoids and flavonols.**

Total phenolic contents of the extracts was determined using the Folin-Ciocalteu method as described by Mohdaly et al. (2010). This assay is based on a green-blue complex arise after oxidation of phenolic groups by phosphomolybdic and phosphotungstic acids (FC reagent) measured at the absorbance of 765 (Barillari et al., 2006). A 20 μl aliquot of extract solution was mixed with 1.58 ml water and 100 μl of Folin-Ciocalteu reagent. The mixture was vortexed for 3–8 minutes and then followed by adding 300 μl of 7.5% Na_2_CO_3_ solution. After vortex for 30 seconds, the mixture was allowed to stand in room temperature for 2 hours. The absorbance was measured at 765 nm against the blank using spectrophotometer (Jenway System, UK). Gallic acid was used as standard for the calibration curve. Total phenolic content expressed as gallic acid equivalent (GAE) was calculated by plotting to the standard calibration curve (Figure 4). The final concentration expressed as mg GAE/g dry weight (DW).

### Total flavanoids

Total flavonoids content of the extracts was determined by the method of Mohdaly et al. (2010). A 0.5 ml aliquot of 20 g/L AlCl_3_ ethanolic solution was added to 0.5 ml of extract solution. Total flavonoid content was determined at 420 nm after standing for 1 hour at room temperature. The presence of flavanoids was indicated by the yellow color development. A freshly prepared quercetin was used as standard. Total flavonoid content was then further expressed as mg quercetin equivalent (QE) per gram dry weight of plant material (mg QE/gDW) and was calculated according to the following linear equation as shown in calibration curves (Figure 4).

### Total flavonols

The concentration of total flavonols was measured according the method of Mohdaly et al. (2010). To 0.5 ml of extract solution, 0.5 ml AlCl_3_ 20 g/L in ethanol and 0.75 ml NaCH_3_COOH 50 g.L^-1^ were added and mixed. The mixture was allowed for 2.5 hours in room temperature. The absorbance at 440 nm was read using semi-cuvet. The total flavonols were calculated as quercetin equivalent (mg QE/gDW) using the following equation based on the regression equation between quercetin standard and absorbance (Figure 4).

### Rosmarinic acid (α-*o*-caffeoyl-3,4-dihydroxyphenyllactic acid)

Estimation of rosmarinic acid content was calculated based on a spectrophotometric method described by Kiong et al. (2008). Briefly, an aliquot of 1 ml of IOSC extract was diluted by the addition of 3 ml of 70% (v/v) methanol and then exactly 1 ml was transferred into semi-cuvet for measurement using spectrophotometer. The production of rosmarinic acid was determined spectrophotometrically at 333 nm (*ε* = 19,000 L/mol/cm). The RA content was calculated and then later expressed as mg/g DW.

### Phenolic acids (reverse-phase HPLC-DAD) analysis

In brief, all elicitation and tea fermentation samples were harvested, weighed and immersed in liquid nitrogen to prevent phenolic compounds volatilization and then were freeze-dried. The lyophilized samples were grounded by flint mill (Retsch GmBH, Haan, Germany) (13416 x *g*, 5 min) to a fine powder. The powder samples were weighed of 20 mg accurately and extracted within 15 minutes using 750 μl 70% methanol in HPLC water (v/v, pH 4.0) in an ultrasonic water bath on ice. Samples were centrifuged for 5 minutes at 13000 x *g*. The supernatants were collected and the pellets were re-extracted twice with 500 μl 70% methanol in HPLC water (v/v, pH 4.0). As an internal standard, cinnamic acid with the volume of 40 μl of 3 mM (diluted in 70% methanol in HPLC water (v/v, pH 4)) was added to the first extraction step. The combined supernatants from each sample were kept in a rotary evaporator (SPD 111V Speed Vac. Concentrator, Thermo Scientific, USA; CVC 3000V, Vacuubrand GmbH, Wertheim, Germany) at 25°C under vacuum to remove the solvent completely. Residues were re-dissolved in 1 ml 40% acetonitrile in HPLC water (v/v). The samples were filtered using 0.22 μm cellulose acetate filters (Corning® Costar® Spin-X® Plastic Centrifuge Tube Filters, Product-catalogue number: CLS8161 and Lot No.: 25410004, Sigma-Aldrich Chemie GmBH, Steinheim, Germany) and were then analyzed with HPLC.

The separation of phenolic compounds was performed on HPLC (UltiMate SR-3000, Dionex, Idstein, Germany), equipped with LPG-3400SD pump, WPS-3000SL automated sample injector, AcclaimPA C16-column (3 μm, 2.1 x 150 mm, Dionex) and DAD-3000 diode array detector (Dionex) and software Chromeleon 6.8. The column was operated at a temperature of 35°C. The mobile phase consisted of 0.1% phosphoric acid in HPLC water (eluent A) and of 40% acetonitrile in HPLC water (v/v, eluent B). A multistep gradient was used for all separations with an initial injection volume of 40 μl and a flow rate of 0.4 ml/min. The multistep gradient was used as follows: 0–1 min: 0.5% B; 1–10 min: 0.5–40% B; 10–12 min: 40% B; 12–18 min: 40–80% B; 18–20 min: 80% B; 20–24 min: 80–99% B; 24–30 min: 99–100% B; 30–34 min: 100–0.5% B; 34–39 min: 0.5% B. Simultaneous monitoring was performed at 254, 290, and 330 nm. Diode array detection was used for the identification of the compounds. Retention times and UV/visible absorption spectra of the peaks were compared with those of the authentic standards. Phenolic acids concentration was calculated using the internal standard peak area as a reference.

### Statistical Analysis

Experimental results concerning this study were reported as means ± standard deviation (S.D.). Data were subjected to analysis of variance (ANOVA) with p-values < 0.05 using SPSS version 17.0 (SPSS Inc. Chicago, IL, USA).

## Abbreviations

IOSC: *In vitro* sprout culture of *Orthosiphon aristatus*
UFT: Unfermented tea
PFT: Partial-fermented tea
FFT: Full-fermented tea.
